# *PLXNC1* and *RDH13* associated with bilateral convergent strabismus with exophthalmus in German Brown cattle

**Published:** 2012-08-09

**Authors:** Steffen Fink, Stefanie Mömke, Ottmar Distl

**Affiliations:** Institute for Animal Breeding and Genetics, University of Veterinary Medicine Hannover, Bünteweg 17p, 30559 Hannover, Germany

## Abstract

**Purpose:**

We performed an association study for bilateral convergent strabismus with exophthalmus (BCSE) in German Brown cattle using single nucleotide polymorphisms (SNPs) located within six positional candidate genes and additional SNPs from bovine SNP databases surrounding these candidate genes. Mutation analyses included synaptotagmin 3 and 5 (*SYT3*, *SYT5*), carnitine palmitoyl-transferase 1C (*CPT1C*) on bovine chromosome 18 (BTA18), and plexin C1 (*PLXNC1*), intracellular suppressor of cytokine signaling-2 (*SOCS2*), and kinesin family member 21A (*KIF21A*) on BTA5.

**Methods:**

For all six candidate genes, we performed cDNA analyses using eye tissues of three BCSE-affected and three unaffected controls and searched the sequences for polymorphisms. Furthermore, we screened a total of 213 SNPs on BTA5 and 136 SNPs on BTA18 from the bovine SNP databases in 29 BCSE-affected German Brown cattle and 23 breed and sex matched controls for association with BCSE. All SNPs detected within the open reading frame (ORF) of the candidate genes and all SNPs from bovine databases putatively associated with BCSE in the detection sample were genotyped in a random sample of 179 BCSE-affected German Brown cows and 161 breed and sex matched controls and tested for association with BCSE.

**Results:**

In total, we detected five novel SNPs within the coding sequence of the candidate genes *PLXNC1* and *KIF21A*. The association analyses for single SNPs and haplotypes in 340 German Brown cattle revealed significant associations for five SNPs with BCSE. Four of these five SNPs were located within *PLXNC1* and *RDH13* and one SNP in the neighborhood of *PLXNC1*. Each one SNP within *PLXNC1* (DN825458:c.168G>T) and *RDH13* (AM930553:c.703C>A) were significantly associated with BCSE after correcting for multiple testing whereas all other SNPs failed this significance threshold. The marker-trait associations for haplotypes confirmed the significant associations with BCSE for both genes, *PLXNC1* and *RDH13*.

**Conclusions:**

The association analyses for single SNPs and haplotypes corroborated the results of the linkage study that the centromeric region of BTA5 and the telomeric end of BTA8 harbor genes responsible for BCSE. Intragenic SNPs of the genes *PLXNC1* and *RDH13* were experiment-wide significantly associated with BCSE and seem to play an important role in the pathogenesis of BCSE.

## Introduction

Bilateral convergent strabismus with exophthalmus (BCSE) is a widespread hereditary defect known in many cattle breeds, e.g., Jersey, German Fleckvieh, German Holstein, and German Brown [1-5]. The incidence of BCSE was estimated at 0.9% in German Brown cattle [2]. Affected animals show a bilateral symmetric protrusion of the eyeballs associated with an anterior-medial rotation of both eyes. The permanent fixation of the eyeballs in this position leads to a convergence of the normally divergent visual axis. The course of the disease is generally progressive. At an advanced stage blindness occurs. The visual disorientation severely impairs affected cattle. Defects in the lateral rectus muscle and the retractor bulbi muscle of the eye or in their appendant nerves (*Nervus abducens* and *Nervus oculomotorius*) are supposed to cause the development of the bilateral convergent strabismus [6]. The first signs of BCSE can appear with an age of 6 months, but mostly the affected animals are not noticed prior first breeding. This eye anomaly is incurable [1].

In a previously performed whole genome scan using non-parametric linkage and haplotype analysis in a total of 159 German Brown cattle, we identified two genomic regions harbouring loci responsible for BCSE on bovine chromosome 5 (BTA5) and BTA18. The most likely location for the BCSE locus on BTA5 was determined between the markers *BP1* (17.29 cM) and *VDR_SNP* (vitamin D [1,25- dihydroxyvitamin D3] receptor) (47.00 cM) on the centromeric region of BTA5 [7]. This region corresponds to a 20.21 Mb interval between 12.34 (*BP1)* and 32.55 Mb (*VDR)* according to *Bos taurus* genome assembly UMD 3.1.

The BCSE locus on BTA18 between the microsatellites *BMS2785* (72.01 cM) and *BM6507* (78.84 cM) identified by linkage analysis was further refined using association and haplotype analysis including 29 single nucleotide polymorphisms (SNPs). Haplotype association refined the BCSE-region to a 6.24 Mb interval on the telomeric end of BTA18. The haplotypes included five intragenic SNPs of the genes *CPT1C* (carnitine palmitoyltransferase 1C), *SYT5* (synaptotagmin 5), *RDH13* (retinol dehydrogenase 13), and *NLRP7* (NLR family, pyrin domain containing 7) [8].

The aim of the present study was to perform an association study using a dense set of SNPs for both identified BCSE-regions on DNA samples from 179 BCSE-affected individuals and 161 controls. The marker set has been supplemented with polymorphisms from candidate genes which might be involved in the pathogenesis of BCSE due to their expression profile, location in the two BCSE regions and known function in human or rodents. Therefore, each three genes from the BCSE region on BTA5 and BTA18 were screened for polymorphisms within their coding sequence. In addition, the SNPs known in positional candidate genes on BTA18 from a previous analysis [8] were also used in the present validation study.

Two of the genes *SYT3* (synaptotagmin 3) and *SYT5* belong to a family of synaptotagmin genes and were both located within the BCSE region on BTA18. Synaptotagmin is a membrane-associated protein that interacts with SNAREs (soluble N-ethylmaleimide-sensitive-factor attachment receptors) which are proteins that act as catalysts for membrane fusion [9,10], phospholipid membranes, Ca^2+^ channels, and other proteins which are involved in the endocytosis process [11-15]. *SYT3* is highly expressed in brain and in various endocrine tissues [16]. The second candidate gene of the synaptotagmin family *SYT5* is expressed in several non-neuronal tissues like kidney, heart, lung, and adipose tissue as well as in brain and PC12 cells (cell line derived from a pheochromocytoma of the rat adrenal medulla) with higher levels [17,18].

The third gene on BTA18, *CPT1C*, is specifically expressed within the endoplasmic reticulum (ER) in neurons of the brain [19] and in the retinal pigment epithelium [20]. CPT1C is believed to regulate the synthesis of sphingolipids and ceramids, which are important for signal transduction, modification of neuronal membranes, and brain plasticity [21-23].

Within the BCSE region on BTA5, we have chosen three candidate genes which might be involved in the pathogenesis of BCSE. *PLXNC1* (Plexin C1) belongs to a subfamily of plexin genes which function as receptors for semaphorins [24]. Semaphorins are a large family of proteins which influence neuronal connectivity, axonal and dentritic growth, guidance, branching and pruning, and synapse formation [25]. PLXNC1 specifically binds semaphorin 7a (Sema7a), a glycosylphosphatidylinisotol (GPI) membrane-associated semaphorin [24,26]. Semaphorin7a promotes central and peripheral axon growth [27]. An expression study in nonneuronal and especially neuronal tissues of rats showed that *Sema7a* and *Plxnc1* were both expressed in multiple neuronal systems of brain, the spinal cord (also motoneurons), in muscles and the eye, particularly the retinal ganglion layer and the inner segment layer, the lens, and the lens epithelium [28].

*SOCS2* (intracellular suppressor of cytokine signaling-2) is a member of the SOCS gene family which is highly expressed in the central nervous system (CNS) during neural development in mouse and also in adult mouse nervous system [29,30].

The sixth candidate gene we analyzed was *KIF21A* (kinesin family member 21A) which is also located on BTA5. KIF21A belongs to a family of plus end-directed kinesin motor proteins. Kinesin motor proteins are used in neurons to transport essential cellular components along axonal microtubules. In human, mutations in the *KIF21A* gene were identified as responsible for congenital fibrosis of extraocular muscles 1 (CFEOM1). CFEOM1 is characterized by variable amounts of restriction of the ocular muscles innervated by the oculomotor and trochlear nerves [31] which leads to progressive bilateral convergent strabismus [32]. Progressive external ophthalmoplegia (PEO), Duane retraction syndrome (DRS) and congenital fibrosis of the extraocular muscles (CFEOM) are diseases in man with similarities in pathology and clinical features to BCSE in cattle. Candidate genes for PEOs and DRS could be ruled out as responsible for BCSE [8] and for CFEOMs, *KIF21A* had been identified as a candidate near to the BCSE region on BTA5.

## Methods

### Animals, phenotypic data and DNA/RNA extraction

We collected blood samples of unrelated as well as BCSE-affected and BCSE-unaffected German Brown cows. The cows unaffected by BCSE were more than six years old. Thus, these animals are very unlikely to develop the BCSE phenotype. Genomic DNA was extracted from EDTA blood samples through a standard ethanol fractionation with concentrated sodiumchloride (6M NaCl) and sodium dodecyl sulfate (10% SDS). Concentration of extracted DNA was determined using the Nanodrop ND-1000 (Peqlab Biotechnologie, Erlangen, Germany). DNA concentration of samples was adjusted to 20 ng/μl. For cDNA analysis, we took biopsies from retina, *N. opticus*, and ocular muscles (*M. rectus lateralis* and *M. retractor bulbi*) of three unaffected and three severely affected cows (BCSE stage 3) [5]. These samples were taken 15–30 min after the cows were slaughtered. Tissue samples were conserved using RNA-later solution (Qiagen, Hilden, Germany). RNA was extracted from the ocular tissues using the Nucleospin RNA II-Kit (Macherey-Nagel, Düren, Germany) and transcribed into cDNA using SuperScript III Reverse Transcriptase (Invitrogen, Karlsruhe, Germany).

### Genotyping

Genotyping was performed in 29 BCSE-affected German Brown cows and 23 breed and sex matched controls on the Sequenom MassARRAY iPLEX Gold system (Sequenom, San Diego, CA) using standard procedures as recommended by the manufacturer. SNPs were selected from bovine SNP databases to cover the candidate regions with dense SNP sets at average distances of 50–100 kb.

Validation of SNPs associated in the detection sample was done in 179 BCSE-affected cows and 161 breed and sex matched controls. These animals were genotyped for 19 SNPs using restriction endonucleases or a 7300 Realtime PCR system (Custom TaqMan® SNP Genotyping Assays; Applied Biosystems, Darmstadt, Germany). For investigation of putative restriction fragment length polymorphisms (RFLPs), NEBcutter V2.0 was used. The sequences for RFLPs (restriction fragment length polymorphisms) were polymerase chain reaction (PCR) -amplified. Two µl of DNA were used as template in the PCR which was performed in 30 µl reaction volumes containing 2 µl (~20 ng/µl) genomic DNA, 3 µl 10× PCR buffer, 6 µl 10× PCR Enhancer (Peqlab Biotechnologie, Erlangen, Germany), 0.6 µl (10 µM) of each primer, 0.6 µl dNTPs (10 mM each), and 0.2 µl (5 U/µl) Taq polymerase (Qbiogene, Heidelberg, Germany). After 5 min initial denaturation at 94 °C, 36 cycles of 45 s at 94 °C, 60 s annealing temperature, and 60 s at 72 °C were performed in TProfessional thermocyclers (Biometra, Göttingen, Germany).

For genotyping the RFLPs, we used 20 µl reaction volumes containing 2 µl buffer, possible 0.2 µl BSA (BSA) dependent on the used endonucleases, and 1.5 U endonucleases with 15 µl of the amplicon. Genotypes were determined by gel electrophoresis using 1.5 or 2% agarose gels dependent on the expected allele sizes and evaluated by visual examination.

The Custom TaqMan® SNP Genotyping Assays contained in 12 µl reactions 6.0 µl SensiMix DNA kit (Quantance, London, UK), 0.3 µl Custom TaqMan® SNP Genotyping Assays and 2 µl template DNA. The reaction was performed on 7300 Realtime PCR system: 10 min initial denaturation at 95 °C, 40 cycles of 15 s at 92 °C, and 60 s at 60 °C.

### Bioinformatic cDNA analysis

We searched expressed sequence tags (ESTs) and the annotated bovine gene by cross-species BLAST searches with the corresponding human reference mRNA sequences for *SYT3* (NM_032298), *SYT5* (NM_003180), *CPT1C* (NM_152359), *PLXNC1* (NM_005761), *SOCS2* (NM_003877) and *KIF21A* (XM_863894) in the database of the National Center for Biotechnology Information (NCBI). Table 1 gives an overview of the structure of the human genes and their orthologs in *Bos taurus*.

**Table 1:** Candidate genes.

** **	** **	***Homo sapiens* (GRCh37.p5)**				***Bos taurus* (UMD 3.1)**				
**Gene**		**HSA**	**DNA (bp)**	**mRNA (bp)**	**Number of exons**	**BTA**	**DNA (bp)**	**mRNA (bp)**	**Protein (aa)**	**Number of exons**
*SYT3*	synaptotagmin III	19	17,859	2913	11	18	12,334	2273	591	9
*SYT5*	synaptotagmin V	19	7252	2612	8	18	6591	1642	386	9
*CPT1C*	carnitine palmitoyltransferase 1C	19	22,624	2910	20	18	19,078	2783	779	19
*PLXNC1*	plexin C1	12	158,953	7346	31	5	152,281	4951	1575	32
*SOCS2*	suppressor of cytokine signaling 2	12	6381	3733	3	5	4880	2172	198	4
*KIF21A*	kinesin family member 21A	12	150,163	6601	37	5	184,440	6456	1653	37

An overview about the structure of the candidate genes *SYT3*, *SYT5*, *CPT1C, PLXNC1, SOCS2*, and *KIF21A* of *Homo sapiens* in comparison to *Bos taurus* is given.

We found the bovine mRNA (XM_580820 and NM_001083744) and three overlapping bovine ESTs for *SYT3* (DY181492, DV883291, and CO874669) and *SYT5* (DY181856, DN536592, and DN517686), which cover 57% and 73% of the human mRNA sequence with an identity of 94.3% and 91.2%, respectively. For *CPT1C*, we found five mostly overlapping ESTs (CR454069, BE664033, CO881322, EH206602, and CK845964), which cover 85% of the human mRNA (NM_152359) with an identity of 87.8% and the bovine mRNA of *CPT1C* (XM_591445). Using BLAST analysis we detected six single bovine ESTs (EH144736, DN825458, AM037678, AW418137, EH152007, and DY167320) which could be aligned to the human mRNA of *PLXNC1* with a total coverage of 74% and an identity of 91.4% and additionally the bovine predicted mRNA sequence (XM_596354). For *SOCS2*, we identified the orthologous bovine mRNA sequence (NM_177523) and three overlapping ESTs which cover 80% of the human mRNA sequence (NM_003877) with an identity of 98.6%. Furthermore, we detected the bovine mRNA of *KIF21A* (XM_863894) and ten mostly overlapping bovine ESTs (EE364333.1, EE239647.1, CV984291.1, DY186213.1, CO883466.1, EH139068.1, DT828764.1, EE340245.1, EW681163.1, and EE907820.1) within the bovine NCBI database. These ESTs covered 97% of the published human mRNA (NM_017641.2) with an identity of 90.8%. We amplified the cDNA sequence corresponding to the open reading frames (ORF) of the six candidate genes. We designed primers using Primer3 as far as possible within the EST sequences, and in the published mRNA sequence of the different genes. These primer sequences can be observed in Appendix 1.

### Sequencing, detection, and genotyping of single nucleotide polymorphisms for the validation study

We used the cDNA of three BCSE-affected and three unaffected German Brown cows and performed PCRs in a total volume of 30 µl. After purification of the PCR products with MinElute 96 UF Plate (Qiagen), the amplicons were directly sequenced with the DYEnamic ET Terminator Cycle Sequencing kit (GE Healthcare, Freiburg, Germany) on a MegaBACE 1000 capillary sequencer (GE Healthcare). Sequence data was analyzed using Sequencher 4.7 (GeneCodes, Ann Arbor, MI).

We analyzed a total of 36 PCR-products within the six candidate genes. We genotyped the cDNA-SNPs of the six candidate genes *PLXNC1*, *KIF21A, CPT1C*, *NLRP7, SYT5*, and *RDH13* for the sample of 179 BCSE-affected German Brown cows and 161 unaffected cows of the same breed with a 7300 Realtime PCR system (Custom TaqMan® SNP Genotyping Assays; Applied Biosystems, Darmstadt, Germany). In addition, all five SNPs on BTA18 found significantly associated with BCSE using a haplotype marker-trait analysis in a previous study [8] were genotyped on the same sample of 340 German Brown cows.

### Statistical analyses

A case-control association analysis based on χ^2^-tests for genotypes, alleles and trend of the alleles was performed using the CASECONTROL procedure of SAS Genetics (SAS, version 9.3; Statistical Analysis System, Cary, NC)]. The ALLELE procedure of SAS was used for estimation of allele frequencies and tests for Hardy–Weinberg equilibrium (HWE) of genotype frequencies. The permutation procedure of PLINK (version 1.07) was used for adaptive permutation approach of the SNPs [33]. Statistical calculation of pairwise LD was performed and pictured using HAPLOVIEW 4.0 [34]. We used the Tagger algorithm r^2^≥0.8 [35] to detect SNPs with strong linkage disequilibrium (LD) among alleles. Subsequently, the association of haplotypes with BCSE was tested using the HAPLOTYPE procedure and the proportion of explained phenotypic variance of the trait was estimated by a multiple ANOVA using the GLM procedure of SAS.

## Results

### Association analysis in the detection sample

Within the BCSE region on BTA5, a total of 213 SNPs were genotyped whereof only four SNPs were not in HWE. The BCSE interval with 6.82 Mb on the telomeric end of BTA18 contained 136 SNPs and nine of them were not in HWE. Each two SNPs were in strong linkage disequilibrium (LD) on both BCSE regions and thus, each one SNP was discarded from BTA5 and 18. The association analyses for 334 SNPs revealed 40 SNPs in the BSCE region on BTA5 and 18 SNPs in the BCSE region on BTA18 associated with BCSE at p-values<0.1 in χ^2^-tests for distribution of genotypes or alleles or in trend tests (data not shown). In the subsequent haplotype and variance analyses, we tested these SNPs to find the sparsest combinations of these SNPs explaining the largest proportion of variance for BCSE and being most significant in haplotype-trait associations.

### Multiple ANOVA and haplotype association

The genotypes of the markers Hapmap42731-BTA-92931, ARS-BFGL-NGS-12640, Hapmap41951-BTA-73168, BTA-73209-no-rs, and ARS-BFGL-NGS-49972 on BTA5 explained the largest proportion of phenotypic variance for BCSE with a value of 59.56%. The marker-trait association including these five SNPs (Hapmap42731-BTA-92931, ARS-BFGL-NGS-12640, Hapmap41951-BTA-73168, BTA-73209-no-rs, and ARS-BFGL-NGS-49972) on BTA5 was significant (χ^2^=34.61, p=0.001). In total, 15 different haplotypes of these markers had a frequency of at least 1% (Table 2). Theses haplotypes spanned the region from 24.46 Mb (Hapmap42731-BTA-92931) to 32.85 Mb (ARS-BFGL-NGS-49972) on BTA5. Four individual haplotypes were significantly associated with the affection status and one of these haplotypes (A-G-G-A-G) occurred with a frequency of 26.7% in our sample. The G-A-A-C-G, A-G-G-C-C and A-A-A-C-G haplotypes were not present in the sample of BCSE-affected cows. These haplotypes occurred with a frequency of 17.2, 6.7 and 5.6% in the sample of controls (Table 2). The marker combination of the four SNPs Hapmap42731-BTA-92931, ARS-BFGL-NGS-12640, BTA-73209-no-rs, and ARS-BFGL-NGS-49972 showed a lower p-value in the marker-trait association test (χ^2^=30.62; p<0.0001) than the haplotype extended by the SNP Hapmap41951-BTA-73168. The proportion of phenotypic variance for BCSE explained by the genotypes of these four markers was 56.4%.

**Table 2:** Haplotype association (BTA5).

**Haplotype**					** **	** **	**Frequency (%)**		** **	** **
**1**	**2**	**3**	**4**	**5**	**Frequency total (%)**	**Standard error**	**Controls**	**Cases**	**χ^2^**	**p**
**A**	**G**	**G**	**A**	**G**	**26.70**	**0.044**	**16.46**	**34.52**	**4.28**	**0.039**
A	G	A	A	G	16.29	0.036	17.27	18.95	0.33	0.564
A	G	A	C	G	10.83	0.031	10.50	8.43	0.35	0.553
G	G	A	A	G	8.13	0.027	7.20	9.05	0.12	0.730
G	G	G	C	G	7.99	0.027	6.92	9.04	0.16	0.691
**G**	**A**	**A**	**C**	**G**	**6.64**	**0.025**	**17.21**	**0**	**12.43**	**<0.001**
G	G	A	C	G	4.92	0.021	5.25	3.22	0.37	0.544
G	A	G	A	G	3.23	0.017	0	5.17	2.23	0.135
G	G	G	A	C	2.88	0.016	4.60	1.56	0.84	0.358
A	A	A	A	C	2.78	0.016	0	3.45	1.41	0.235
**A**	**G**	**G**	**C**	**C**	**2.18**	**0.014**	**6.68**	**0**	**5.65**	**0.018**
**A**	**A**	**A**	**C**	**G**	**1.92**	**0.014**	**5.61**	**0**	**4.44**	**0.035**
A	G	G	A	C	1.65	0.013	0	1.89	0.79	0.374
G	G	G	A	G	1.49	0.012	0	2.99	1.58	0.209
G	A	A	A	G	1.23	0.011	0	1.72	0.69	0.407

Frequencies of the haplotypes with at least 1% in the sample of 52 German Brown cattle and their standard errors, haplotype frequencies of cases and controls and their associations with BCSE on bovine chromosome 5 are shown. In the haplotype column, 1=Hapmap42731-BTA-92931; 2=ARS-BFGL-NGS-12640; 3=Hapmap41951-BTA-73168; 4=BTA-73209-no-rs; 5=ARS-BFGL-NGS-49972.

On the telomeric end of BTA18, the markers ARS-BFGL-NGS-93837, Hapmap42211-BTA-43910, ARS-BFGL-BAC-31654, ARS-BFGL-NGS-1786, and ARS-BFGL-NGS-41595 were found to contribute the largest proportion of phenotypic variance for BCSE. These five markers reached 52.10% of the total phenotypic variance. The haplotypes composed of these five SNPs reached significant results in marker-trait associations (χ^2^=34.12, p-value=0.001). There were eleven different haplotypes that had a frequency of at least 1% (Table 3). Three individual haplotypes were significantly associated with the BCSE-affection status and occurred with a frequency of more than 5% in our sample. The A-G-A-A-A haplotype could be assigned clearly to the BCSE-affected animals (8.5%) because none of the controls showed this individual haplotype. The G-G-A-A-A haplotype was found with 21.6% in the sample of unaffected animals and with 3.7% in BCSE-affected animals. The third associated haplotype (G-G-A-G-A) was present in 27.3% of the BCSE-affected animals and occurred with 7.4% in the sample of controls. One additional individual haplotype (G-G-G-G-A) failed the threshold of significance with a p-value of 0.06. This haplotype was present in 10.0% of the controls and in 24.7% of the BCSE-affected animals (Table 3).

**Table 3:** Haplotype association (BTA18).

**Haplotype**					** **	** **	**Frequency (%)**		** **	** **
**1**	**2**	**3**	**4**	**5**	**Frequency total (%)**	**Standard error**	**Controls**	**Cases**	**χ^2^**	**p**
**G**	**G**	**A**	**G**	**A**	**23.01**	**0.041**	**7.40**	**27.26**	**6.92**	**0.009**
G	G	G	G	A	18.31	0.038	10.03	24.68	3.68	0.055
G	G	A	G	G	17.12	0.037	25.87	13.34	3.07	0.080
**A**	**G**	**A**	**A**	**A**	**7.75**	**0.026**	**0**	**8.59**	**3.92**	**0.048**
**G**	**G**	**A**	**A**	**A**	**7.68**	**0.026**	**21.63**	**3.69**	**13.93**	**<0.001**
G	G	G	A	G	6.41	0.024	7.79	6.90	0.17	0.680
G	G	A	A	G	3.41	0.018	5.29	0	2.54	0.111
A	G	G	A	A	3.31	0.018	4.35	2.92	0.18	0.670
A	G	G	G	A	3.18	0.017	0	5.28	2.34	0.126
G	A	A	A	A	2.37	0.015	1.99	0	1.44	0.231
A	G	A	G	G	1.07	0.010	0	1.86	0.84	0.361

Frequencies of the haplotypes with at least 1% in the sample of 52 German Brown cattle and their standard errors, haplotype frequencies of cases and controls and their associations with BCSE on bovine chromosome 18 are shown. In the haplotype column, 1=ARS-BFGL-NGS-93837; 2=Hapmap42211-BTA-43910; 3=ARS-BFGL-BAC-31654; 4=ARS-BFGL-NGS-1786; 5=ARS-BFGL-NGS-41595.

### Mutation analysis of candidate genes in the bovine *SYT3*, *SYT5*, and *CPT1C* on BTA18 and *PLXNC1*, *SOCS2*, and *KIF21A* on BTA5

We revealed a total of five exonic SNPs within the six candidate genes (Table 4) which were chosen due to their expression profile, known function in other species and their location on BTA5 and 18, respectively.

**Table 4:** Single marker analysis.

			**Number of**		**MAF (%)**				
**SNP ID**	**Chromosome- position (bp)**	**Location (gene name) UMD 3.1**	**Controls**	**Cases**	**Controls**	**Cases**	**HET (%)**	**PIC (%)**	**p (HWE)**
Hapmap42731-BTA-92931	5–21768260	intergenic	161	179	39.94	34.32	45.12	35.81	0.535
**ARS-BFGL-NGS-12640**	**5–23861315**	**intergenic**	**159**	**178**	**19.28**	**11.01**	**23.08**	**22.17**	**0.100**
DN825458:c.168G>T*	**5–24073205**	**PLXNC1**	**160**	**177**	**31.37**	**43.49**	**51.39**	**35.96**	**0.093**
XM_596354:c.1678A>G*	5–24142953	PLXNC1	160	177	26.97	24.40	37.96	30.94	0.889
EH152007:c.462T>C*	5–24215836	PLXNC1	159	177	22.73	22.92	36.53	29.08	0.538
Hapmap41951-BTA-73168	5–28442563	Intergenic	160	179	43.79	42.31	51.38	37.02	0.391
BTA-73209-no-rs	5–29496625	DIP2B	161	179	36.04	31.66	47.56	34.75	0.260
ARS-BFGL-NGS-49972	5–30012017	intergenic	160	179	12.01	7.40	18.65	15.89	0.196
AM931450:c.205T>G*	5–42079372	KIF21A	111	137	55.41	42.86	57.0	37.5	0.211
ARS-BFGL-NGS-93837	18–55807264	MAMSTR	160	179	14.38	16.86	28.44	23.02	0.194
AM930539:g.569A>G	18–56565243	CPT1C	160	179	25.00	30.95	38.57	32.24	0.386
Hapmap42211-BTA-43910	18–58203733	intergenic	160	179	9.80	15.32	14.72	12.71	0.151
ARS-BFGL-BAC-31654	18–62250437	intergenic	159	179	39.22	34.32	45.57	35.62	0.751
ARS-BFGL-NGS-1786	18–62571431	intergenic	160	176	31.17	32.93	46.60	34.15	0.231
AM930544:g.71G>A	18–62704882	SYT5	160	179	8.75	6.51	14.45	12.92	0.448
**AM930553:c.703C>A**	**18–62800146**	**RDH13**	**160**	**179**	**19.02**	**9.88**	**23.90**	**21.64**	**0.544**
**AM930547:g.194C>T**	**18–62800898**	**RDH13**	**160**	**179**	**17.50**	**9.52**	**23.71**	**20.71**	**0.839**
**AM930543:g.103T>G**	**18–62878596**	**NLRP7**	**160**	**179**	**31.74**	**39.08**	**43.40**	**35.29**	**0.319**
ARS-BFGL-NGS-41595	18–63400996	MBOAT7	159	178	34.74	35.50	41.72	35.24	0.118

Shown are the single nucleotide polymorphisms (SNPs, n=19) which were genotyped for the sample of 340 German Brown cows, their location according to UMD 3.1, their number of genotyped cases and controls, minor allele frequencies (MAF) of genotyped cases and controls, polymorphism information content (PIC), heterozygosity (HET) and test results for Hardy–Weinberg equilibrium (HWE). SNPs detected by cDNA sequencing are marked by an asterisk.

Within the coding sequence of *SYT3* and *CPT1C* which were located in the proximal BCSE region on BTA18 and in the neighborhood of the significantly associated Hapmap42211-BTA-43910 SNP (Table 4), no polymorphisms could be detected. The third candidate gene *SYT5* is located 140 kb next to the microsatellite *DIK5109* which reached the highest values for Zmean and LOD score in linkage analysis on BTA18 [7]. This gene did also not harbor any SNP in the complete coding sequence.

*PLXNC1* and *SOCS2* are located closely to the ARS-BFGL-NGS-12640 SNP which reached significant results in allele, genotype and trend test statistics. We did not detect a polymorphism located within the coding sequence of the *SOCS2* gene. Within the coding sequence of *PLXNC1*, we identified three SNPs (Table 4). A G>T SNP (DN825458:c.168G>T) is located at position 930 bp of bovine mRNA in exon 1. This G>T transversion is a synonymous mutation. The second SNP which results in an amino acid exchange from threonine to alanine (p.Thr308Ala), was found at position 115 of exon 6 (XM_596354:c.1678A>G). This A>G transition changes a GCG triplet to a GCT triplet. This means there is a change from a polar and uncharged amino acid with a hydroxyl group to an unpolar amino acid. In addition, we detected a synonymous SNP in exon 27 (EH152007:c.462T>C).

Most of the cDNA sequences of eye tissues perfectly matched to the published bovine mRNA. In all analyzed eye-tissues only three additional consecutive base pairs (XM_863894.2:c.4107insTAG) were detected in the cDNA sequence of *KIF21A*. This is supposed to be caused by an alternative splicing at the 5′ splice site of intron 29 of *KIF21*A, which does not result in a frameshift. The only consequence is the insertion of an additional amino acid (XP_868987.2:p.His1103_Arg1104insSer) into the primary protein sequence. Both splice variants occurred equally in the three severely BCSE-affected and the three unaffected cows. All tested animals showed both splice variants of cDNA. Furthermore, we detected two exonic SNPs in the ORF of *KIF21A*. One SNP was found within exon 13 (AM931451:c.292A>G), but this polymorphism has no obvious effect on the amino acid sequence. It was only present in two control animals. Therefore, we skipped this SNP for further analysis due to the low allele frequency in our sample. A further SNP within exon 6 of *KIF21A* (AM931450:c.205T>G) causes an amino acid exchange from isoleucin to serin. This means an exchange from an unpolar hydrophobic amino acid to a polar, uncharged and hydrophilic amino acid in primary structure of the protein product. All SNPs detected using cDNA analysis of candidate genes were not in LD. The pairwise r^2^-values between the SNP alleles on BTA5 were very low.

### Validation of single marker associations

Validation was performed for each five SNPs composing significant haplotypes on the BCSE regions on BTA5 and 18, respectively, and in addition, for the candidate gene-associated SNPs of the BCSE regions. These SNPs were located within the genes *PLXNC1* and *KIF21A* on BTA5 and within *CPT1C*, *SYT5*, *NLRP7*, and *RDH13* on BTA18. The intragenic BTA18-SNPs included in the present analysis had been shown in a previous study to compose a significant BCSE-associated haplotype [8]. Results of the case-control analysis of the 19 SNPs in a sample of 179 BCSE-affected and 161 controls are shown in Table 5.

**Table 5:** Single marker association in the validation sample of 340 German Brown cows.

**SNP ID**	**Chromosome - position (bp)**	**χ^2^ allele**	**p allele**	**χ^2^ genotype**	**p genotype**	**χ^2^ trend**	**p trend**
Hapmap42731-BTA-92931	5–21768260	2.34	0.126	4.64	0.098	2.26	0.132
**ARS-BFGL-NGS-12640**	**5–23861315**	**6.73**	**0.010**	**8.07**	**0.017**	**6.36**	**0.011**
DN825458:c.168G>T*	**5–24073205**	**17.18**	**<0.001**	**20.64**	**<0.001**	**19.24**	**<0.001**
XM_596354:c.1678A>G*	5–24142953	0.54	0.460	0.59	0.742	0.54	0.461
EH152007:c.462T>C*	5–24215836	<0.001	0.996	0.60	0.739	<0.001	0.996
Hapmap41951-BTA-73168	5–28442563	0.17	0.681	0.62	0.732	0.18	0.673
BTA-73209-no-rs	5–29496625	1.35	0.243	2.60	0.272	1.45	0.228
ARS-BFGL-NGS-49972	5–30012017	3.20	0.07	3.81	0.149	3.46	0.063
AM931450:c.205T>G*	5–42079372	2.48	0.115	2.90	0.235	2.88	0.090
ARS-BFGL-NGS-93837	18–55807264	0.91	0.338	1.85	0.396	0.98	0.321
AM930539:g.569A>G+	18–56565243	2.17	0.141	2.26	0.323	2.07	0.150
Hapmap42211-BTA-43910	18–58203733	2.38	0.123	2.58	0.108	2.58	0.108
ARS-BFGL-BAC-31654	18–62250437	1.38	0.238	1.74	0.419	1.36	0.242
ARS-BFGL-NGS-1786	18–62571431	0.14	0.702	0.67	0.715	0.16	0.692
AM930544:g.71G>A+	18–62704882	1.87	0.172	3.86	0.146	1.94	0.163
**AM930553:c.703C>A+**	**18–62800146**	**9.24**	**0.005**	**9.22**	**0.002**	**8.95**	**0.003**
**AM930547:g.194C>T+**	**18–62800898**	**8.06**	**0.006**	**8.42**	**0.015**	**8.15**	**0.004**
**AM930543:g.103T>G+**	**18–62878596**	**6.18**	**0.013**	**7.15**	**0.028**	**5.88**	**0.015**
ARS-BFGL-NGS-41595	18–63400996	0.01	0.900	0.04	0.979	0.01	0.903

The results of association analysis for 19 SNPs on BTA5 and BTA18 with bilateral convergent strabismus with exophthalmus in German brown cattle, their χ^2^-test statistics of the case-control analysis and p-values (p) are presented. Five SNPs detected by cDNA analyses are marked by an asterisk and five SNPs that composed the associated haplotype on BTA18 in a previous study [8] are marked with a plus sign.

On BTA5, the intergenic ARS-BFGL-NGS-12640 SNP reached significant results in genotype, allele and trend test statistics. This SNP was significantly associated with BCSE (χ^2^-values at 6.36-8.07, p-values at 0.01-0.02). The highest association was found for the intragenic *PLXNC1* DN825458:c.168G>T SNP with χ^2^-values at 17.2–20.6 and corresponding p-values at 1.15^−5^-3.40^−5^. All other seven BTA5 SNPs showed no significant results.

On BTA18, the SNPs AM930543:g.103T>G (χ^2^-values at 5.9–7.2 with p-values at 0.013- 0.028), AM930547:g.194C>T (χ^2^-values at 8.1–8.4 with p-values of 0.004–0.015) and AM930553:c.703C>A (χ^2^-values at 9.0–9.2 with p-values of 0.002–0.01) showed significant associations. All other BTA18 SNPs were not significantly associated with BCSE (Table 5).

After accounting for multiple testing of 19 SNPs using a Bonferroni correction, only the SNPs DN825458:c.168G>T (p-value<0.001) within *PLXNC1* and AM930553:c.703C>A (p-value<0.05) within *RDH13* reached significant associations with BCSE in German Brown cattle.

### Validation of haplotype association

The haplotype including the SNPs Hapmap 42731-BTA-92931 and DN825458:c.168G>T gave the highest marker-trait associations with a χ^2^-value of 23.61 (p-value<0.0001; Table 6). All four individual haplotypes composed of these SNPs were significantly associated with BCSE and three individual haplotypes reached frequencies >25%. The A-T haplotype occurred with a frequency of 37.6% in all cows genotyped and in the controls and cases with frequencies of 31.3 and 43.2% (χ^2^-value=11.3, p-value<0.001). The further two significantly associated haplotypes G-G and A-G had frequencies of 39.0 and 29.7% in controls and frequencies of 32.1 and 21.8% in BCSE-affected animals.

**Table 6:** Haplotype association for SNPs on BTA5.

** **	** **	** **	**Frequency (%)**		** **	** **
**Haplotype**	**Frequency (%)**	**Standard error**	**Controls**	**Cases**	**χ^2^**	**p**
A-G	25.64	1.59	29.73	21.84	6.09	0.014
A-T	37.56	1.77	31.30	43.23	11.33	<0.001
G-G	35.31	1.75	38.97	32.07	3.90	0.048
G-T	1.49	0.04	2.86	1.49	10.38	0.001

Frequencies of the haplotypes with their frequencies in the total sample of 340 German Brown cattle, their standard errors, haplotype frequencies of cases and controls and their associations with BCSE on bovine chromosome 5 are shown. In the haplotype column, first SNP=Hapmap42731-BTA-92931; second SNP=DN825458:c.168G>T.

Using only SNPs proximal or distal to *PLXNC1*, the haplotype analysis gave no significant result for the proximal SNPs Hapmap 42731-BTA-92931 and ARS-BFGC-NGS12640 (χ^2^=value=7.60, p-value=0.06) or a significant result with a χ^2^-value of 18.9 (p-value=0.009) for the three distal SNPs Hapmap41951-BTA-73168, BTA-73209-no-rs and ARS-BFGL-NGS-49972. The haplotype combined of these five SNPs on BTA5 failed the threshold of significance (χ^2^=41.62, p-value=0.096).

On BTA18, the marker-trait association of all different combinations of the five SNPs (ARS-BFGL-NGS-93837, Hapmap42211-BTA-43910, ARS-BFGL-BAC-31654, ARS-BFGL-NGS-1786, and ARS-BFGL-NGS-41595) failed the significance threshold in all tests. The haplotype containing all five intragenic SNPs (AM930539:g.569A>G, AM930544:g.71G>A, AM930553:c.703C>A, AM930547:g.194C>T, and AM930543:g.103T>G) was not significant in marker-trait association. However, the haplotypes including the SNPs AM930547:g.194C>T and AM930553:c.703C>A located within *RDH13* reached significant results (χ^2^-value=10.44, p-value=0.015) in the marker-trait associations. Two of the four individual haplotypes composed of these two SNPs (AM930547:g194C>T and AM930553:c.703C>A) reached frequencies >2% (Table 7). These two haplotypes were significantly associated with BCSE. The C-C haplotype occurred with a frequency of 84.6% in all cows genotyped and in the controls and cases with frequencies of 80.3 and 88.5%, respectively (χ^2^-value=9.6, p-value=0.002). The further significantly associated haplotype T-A had frequencies of 16.8 and 10.6% in controls and BCSE-affected animals. In addition, the marker-trait associations of the SNPs AM930543:g.103T>G and AM930553:c.703C>A (χ^2^-value=12.28, p-value=0.007) and the SNP-haplotypes of AM930543:g.103T>G and AM930547:g194C>T (χ^2^-value=11.01, p-value=0.012) reached significant results.

**Table 7:** Haplotype association for SNPs within RDH13 on BTA18.

** **	** **	** **	**Frequency (%)**		** **	** **
**Haplotype**	**Frequency (%)**	**Standard error**	**Controls**	**Cases**	**χ^2^**	**p**
C-A	1.24	0.04	1.91	0. 62	2.53	0.112
C-C	84.59	1.32	80.32	88.49	9.59	0.002
T-A	13.56	1.25	16.84	10.59	6.23	0.013
T-C	0.61	0.03	0.94	0.30	1.27	0.260

Frequencies of the haplotypes with their frequencies in the total sample of 340 German Brown cattle, their standard errors, haplotype frequencies of cases and controls and their associations with BCSE on bovine chromosome 18 are shown. In the haplotype column, first SNP=AM930547:g.194C>T; second SNP=AM930553:c.703C>A.

## Discussion

The association analyses of both BCSE regions on BTA5 and BTA18 for single SNPs and haplotypes revealed the highest significantly associated SNPs with BCSE within the candidate genes *PLXNC1* (BTA5) and *RDH13* (BTA18). Haplotype analyses only including proximally and distally located SNPs of these candidate genes did not result in significant marker-trait test statistics or marker-trait test statistics with higher p-values. Therefore, it is most likely that these candidate genes or nearby located structural mutations may be responsible for BCSE in German Brown cows.

The SNPs within the coding sequences of *PLXNC1* and *RDH13* can be ruled out as causative for BCSE because these polymorphisms did not perfectly match with the phenotypes. The other candidate genes (*SYT3*, *SYT5*, *KIF21A, CPT1C*, and *NLRP7*) are unlikely to harbour polymorphisms causal for this eye anomaly because we did not find significantly associated SNPs at the nominal or experimentwise level for significance. In addition, haplotype associations did not support these candidate genes. To test for potential associations in SNPs surrounding the gene *PLXNC1* and *RDH13*, we employed haplotype analyses. We were not able to demonstrate haplotypes containing SNPs from the candidate gene flanking regions that increased significance of the haplotype association. Robustness of the haplotype analyses was furthermore confirmed when the surrounding haptotypes were extended with the intragenic *PLXNC1* or *RDH13* SNPs. The extended haplotypes reached higher χ^2^-values and lower p-values compared to the haplotypes without these intragenic SNPs. In conclusion, association analyses in this large sample of German Brown cows are supporting *PLXNC1* and *RDH13* as the most likely genes that might harbour a causal mutation for BCSE. To detect these mutations, sequencing of all introns, UTRs and promotors of *PLXNC1* and *RDH13* has to be performed. Particularly, *PLXNC1* has to be considered due to the highest association of all SNPs tested in the present study. *PLXNC1* comprises 32 exons and 152.3 kb genomic sequence. Because of a missing structural variant of the coding sequence in BCSE-affected cattle, we assume that the causal mutation influences the expression level of *PLXNC1* or prevents translation to a functional protein. Evaluation of protein expression would be a possibility to discriminate among the possible mechanisms.

## Appendix 1. cDNA PCR primers.

The PCR primers for amplification of the cDNA of the bovine *SYT3*, *SYT5*, *CPT1C, PLXNC1*, *SOCS2*, and *KIF21A *genes are shown. To access the data, click or select the words “Appendix 1.” This will initiate the download of a compressed (pdf) archive that contains the file.
